# Gallium-68-Labeled Fibroblast Activation Protein Inhibitor as an Alternative Radiotracer to Fluorine 18-Fluorodeoxyglucose: A Case Report of Rare Pulmonary Colloid Adenocarcinoma Diagnosed by PET/CT

**DOI:** 10.7759/cureus.56173

**Published:** 2024-03-14

**Authors:** Areen Mansour, Shahed Obeidat, Dhuha Al-Adhami, Taher Abu Hejleh, Akram Al-Ibraheem

**Affiliations:** 1 Department of Nuclear Medicine and PET/CT, King Hussein Cancer Center (KHCC), Amman, JOR; 2 Department of Medicine, King Hussein Cancer Center (KHCC), Amman, JOR

**Keywords:** fapi pet-ct, 68ga-fapi, pet/ct, pulmonary mucinous adenocarcinoma, 18f-fdg, rare neoplasm

## Abstract

Colloid pulmonary adenocarcinoma represents a seldom encountered neoplasm in clinical practice. The diagnostic process for this rare neoplasm is complicated by its infrequency and the limited understanding of its specific molecular imaging characteristics. We report a 65-year-old male who was diagnosed with pulmonary colloid mucinous cystadenocarcinoma. Fluorine 18-fluorodeoxyglucose (18F-FDG) positron emission tomography/computed tomography (PET/CT) was conducted for initial evaluation. The scan showed mild 18F-FDG expression at the primary tumor site, and several non-18F-FDG-avid mediastinal and paraesophageal lymph nodes exhibited suspicious morphologic features. Owing to the ongoing atrial fibrillation, initial histopathological confirmation of the primary tumor mass carries a sense of risk, prompting the imperative for cardiological assessment before proceeding. Instead, Gallium-68-labeled fibroblast activation protein inhibitor (68Ga-FAPI) PET/CT was performed, expecting this to be more informative in terms of malignancy potential than 18F-FDG PET in colloid mucinous histology. A scan revealed moderate 68Ga-FAPI expression at the primary tumor site but unremarkable 68Ga-FAPI expression at the questionable lymph node. Subsequently, a biopsy from a mediastinal node (left para-aortic) lymph node via endobronchial ultrasound (EUS) showed benign findings. The patient was treated with concurrent chemoradiation. This case underscores the vital role that 68Ga-FAPI PET/CT can play in specific cases of rare cancers, especially when invasive testing for tissue biopsy is not feasible.

## Introduction

Rare cancers, put together, can amount to approximately 22% of all cancers diagnosed worldwide [1]. The diagnosis, staging, management, and treatment of these cancers can be challenging [1]. Mucinous adenocarcinoma, a rare type of lung cancer, has distinct immunohistochemical and molecular characteristics [2]. Positron emission tomography/computed tomography (PET/CT) has evolved as a crucial modality for detecting and evaluating the majority of pulmonary neoplasms, with fluorine 18-fluorodeoxyglucose (18F-FDG) being the standard modality of choice [3]. However, its ability to diagnose and monitor some rare cancers, such as mucinous adenocarcinoma, has been limited due to their biochemical characteristics [1,4,5].

The utilization of the Gallium-68-labeled fibroblast activation protein inhibitor (68Ga-FAPI) radiotracer, which is a transmembrane peptidase that is upregulated in cancer-associated fibroblasts (CAFs), has the potential to address the difficulty posed by tumors with low 18F-FDG avidity [6,7]. Indeed, several research studies have explored the potential use of 68Ga-FAPI for the detection and assessment of rare malignancies [8,9]. Another acknowledged advantage of this novel imaging technique is that it can alleviate the need for extensive patient preparation, such as the dietary restrictions required for 18F-FDG [8].

This case report presents an instance of pulmonary colloid mucinous adenocarcinoma, in which 68Ga-FAPI imaging indicated benign mediastinal lymph nodes that appeared suspicious for metastasis in radiographic evaluation. However, subsequent pathological examination through endoscopic biopsy confirmed the absence of metastasis in these nodes.

## Case presentation

**Figure 1:**
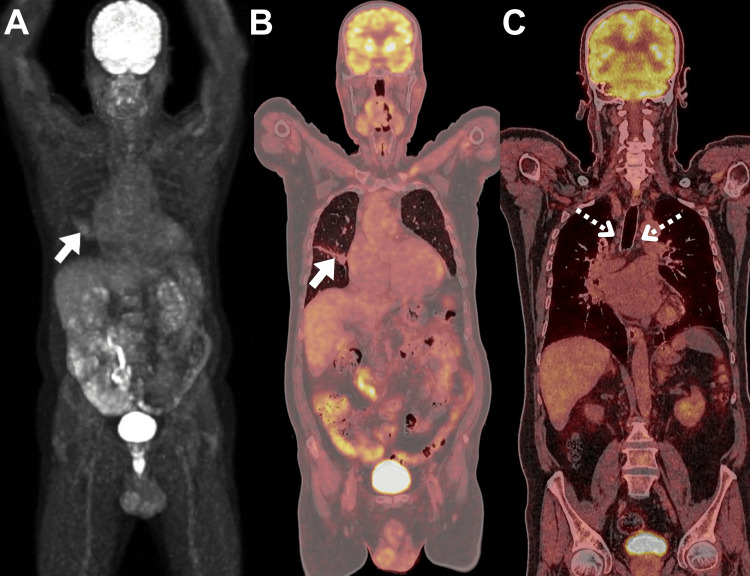
18F-FDG PET/CT images of the patient. (A) MIP, and (B, C) coronal views of 18F-FDG PET/CT images obtained for initial staging. The scan revealed evidence of a mildly hypermetabolic right pulmonary mass (SUVmax of 2.8; arrows), comparable to that of the liver reference. This was associated with multiple non-18F-FDG-avid mediastinal lymph nodes (SUVmax of 1.7; dotted arrows). MIP: maximal intensity projection; 18F-FDG PET/CT: fluorine 18-flurodeoxyglucose positron emission tomography/computed tomography; SUV_max_: maximum standardized uptake value.

A 65-year-old male with multiple comorbidities, including atrial fibrillation, was suspected to have lung malignancy after a CT finding of the right hilar mass. A definitive diagnosis of pulmonary colloidal cystadenocarcinoma was established through a bronchial biopsy. Initial staging was conducted using 18F-FDG PET/CT. Imaging analysis revealed a mildly hypermetabolic right pulmonary mass, consistent with the biopsy-proven primary colloidal cystadenocarcinoma (Figure 1).

**Figure 2:**
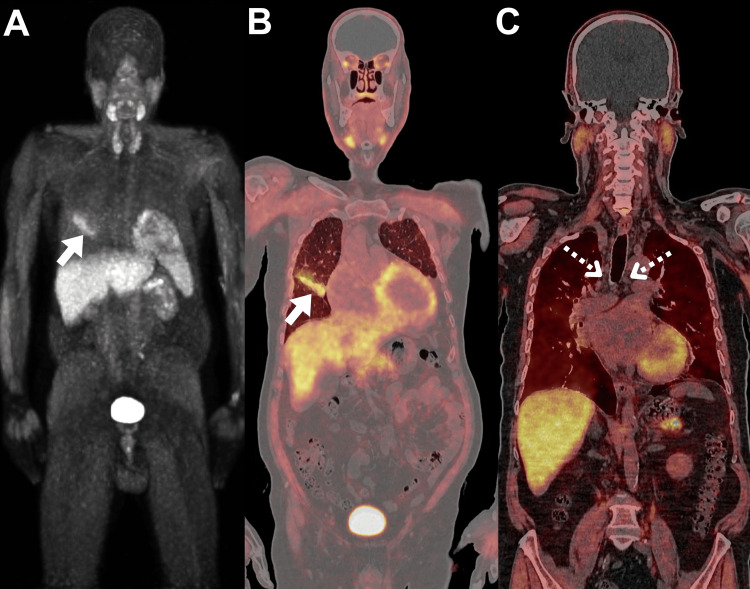
68Ga-FAPI PET/CT images of the patient. (A) MIP, and (B, C) coronal views of 68Ga-FAPI PET/CT images were obtained five days after initial 18F-FDG PET/CT scanning. The scan revealed the same right pulmonary mass with significant 68Ga-FAPI expression (SUVmax of 8.7; arrows). Notably, the previously observed bilateral mediastinal lymph nodes were unremarkable (SUVmax of 1.4, dotted arrows). MIP: maximal intensity projection; 68Ga-FAPl PET/CT: 68Ga-fibroblast activation protein inhibitor positron emission tomography/computed tomography; SUV_max_: maximum standardized uptake value.

Additionally, several enlarged mediastinal and paraesophageal lymph nodes with insignificant 18F-FDG expression were observed. For treatment decisions, it was crucial to have better insight into whether the lymph nodes were malignant. Due to multiple comorbidities and worsening atrial fibrillation, the patient was considered unfit for an invasive lymph node biopsy at this time. Thus, comprehensive whole-body imaging with 68Ga-FAPI PET/CT was pursued because this approach might provide additional information about the nature of the involved lymph nodes (Figure 2).

68Ga-FAPI PET/CT revealed significant 68Ga-FAPI expression in the identified right pulmonary mass; however, the lymph nodes showed insignificant 68Ga-FAPI expression. Subsequent control of atrial fibrillation was established after cardiology consultation, and an endobronchial ultrasound-guided biopsy of the left para-aortic lymph node was conducted. The result showed non-malignant findings in agreement with the 68Ga-FAPI PET/CT findings.

## Discussion

In this case of pulmonary colloid mucinous adenocarcinoma, a rare type of lung cancer, 68Ga-FAPI was more useful than FDG for excluding metastases to mediastinal lymph nodes that were otherwise morphologically enlarged and suspicious on CT scans. 18F-FDG PET/CT is limited by its relatively low avidity in this type of cancer [1,4,5], as the overall sensitivity of 18F-FDG PET/CT for detecting mucinous neoplasms is approximately 59% [4].

68Ga-FAPI is a new tracer that has shown promise in imaging different types of cancer [8,10]. Currently, 68Ga-FAPI has been predominantly utilized and validated for the evaluation of malignancies of hepatobiliary, gynecologic, and gastrointestinal origins [11-14]. The utilization of 68Ga-FAPI PET/CT in these particular tumor types has been shown to yield clinically advantageous tracer kinetics and higher tumor-to-background ratios than 18F-FDG [11-14]. Moreover, in certain instances of uncertainty or when other radiotracers prove inadequate in accurately revealing the disease state, this novel technique has been found to be useful [15,16].

68Ga-FAPI is overexpressed by CAFs, and 90% of all epithelial carcinomas exhibit high expression of FAPI [8]. Most rare tumors originate from epithelial tissue, such as mucinous bronchoalveolar carcinoma [17]. High FAPI expression in mucinous carcinoma predicts aggressive behavior, an increased chance of metastasis, and even resistance to chemotherapy by upregulating the Yes-associated protein 1 (*YAP1*) gene. Therefore, the “FAPI status” of these tumors might be vital for optimal management. In a cohort of various tumors, including uterine sarcoma, appendiceal carcinoma, and thymus cancer, 68Ga-FAPI PET/CT changed the tumor, node, metastasis (TNM) stage in approximately 40% of patients, most of whom were upstaged, and 56.3% of the patients experienced a change in management plan [10]. To our knowledge, this is the first case in which 18F-FDG and 68Ga-FAPI radiotracers were used in the staging of pulmonary colloidal mucinous cystadenocarcinoma, the results of which were concordant with the pathological findings.

## Conclusions

Our results present strong evidence of the additional advantages associated with the utilization of 68Ga-FAPI PET/CT in the assessment of uncommon neoplasms exhibiting low levels of 18F-FDG uptake. In addition, this is the initial instance in which pulmonary colloid mucinous adenocarcinoma was identified using both 18F-FDG and 68Ga-FAPI radiotracers, thereby illustrating the superiority of 68Ga-FAPI in this particular context.
